# Microbiome-Metabolomics Insights into the Milk of Lactating Dairy Cows to Reveal the Health-Promoting Effects of Dietary Citrus Peel Extracts on the Mammary Metabolism

**DOI:** 10.3390/foods11244119

**Published:** 2022-12-19

**Authors:** Yuchao Zhao, Shiqiang Yu, Shuyue Zhang, Yuqin Li, Yan Tu, Ming Liu, Linshu Jiang

**Affiliations:** 1 Beijing Key Laboratory of Dairy Cow Nutrition, College of Animal Science and Technology, Beijing University of Agriculture, Beijing 102206, China; 2 Beijing Beinong Enterprise Management Co., Ltd., Beijing 102206, China; 3 College of Animal Science and Technology, China Agricultural University, Beijing 100183, China; 4 Institute of Feed Research, Chinese Academy of Agricultural Sciences, Beijing 100081, China

**Keywords:** citrus flavonoids, dairy cows, mammary gland, milk bacterial community, milk metabolomics

## Abstract

The effects of dietary supplementation with citrus peel extract (CPE) on milk biochemical parameters, milk bacterial community, and milk metabolites were evaluated. Eight lactating cows were allocated to a replicated 4 × 4 Latin square. Experimental treatments included the control diet (CON), and CON supplemented with CPE at 50 g/d (CPE50), 100 g/d (CPE100), and 150 g/d (CPE150). Supplementing with CPE linearly decreased milk interleukin-6 and malondialdehyde concentrations and linearly increased lysozyme activity and 1,1-diphenyl-2-picrylhydrazyl radical scavenging activity. Compared with CON, the milk of CPE150 cows had fewer abundances of several opportunistic pathogens and psychrotrophic bacteria, such as *Escherichia-Shigella*, *Sphingobacterium*, *Alcaligenes*, *Stenotrophomonas*, and *Ochrobactrum*. Supplementing with CPE significantly altered the metabolic profiling in the milk. The metabolites of flavonoids were enriched in the milk of cows fed CPE150, while some proinflammation compounds were decreased compared with CON. Correlation analysis showed that the change in the bacterial community might partly contribute to the alteration in the expression of milk cytokines. In conclusion, CPE exerts health-promoting effects (e.g., antioxidant, anti-microbial, and anti-inflammatory) in the mammary metabolism of cows due to its flavonoid compounds, which also provide additional value in terms of milk quality improvement.

## 1. Introduction

Bovine milk or dairy products are an important part of the diet of many people worldwide, and global demand for milk is expected to increase as the population increases. However, milk production challenges the metabolism and health of dairy cows. Mammary metabolic stress arises with various effects on the immune system, milk yield, dairy product quality, and the overall well-being of dairy cows [1,2]. The apparently abundant use of antibiotics for controlling mastitis may affect the sales of dairy products [3]. The risk of potential antibiotic residues in milk and antibiotic resistance in animals are increasingly considered serious public health concerns. More recently, as alternatives to antibiotics, phytochemicals have gained increasing attention for their health-promoting effects in lactating dairy cows [4,5].

Flavonoids are ubiquitous phytochemicals found in plants with a wide array of exploitable activities, including antioxidant, anti-inflammatory, and anti-microbial activities [6]. The high volume of peel produced by citrus by-product processing could provide significant quantities of flavonoids. A variety of flavonoid compounds are found in citrus peel, including flavanones (such as hesperidin and naringin) and O-polymethoxylated flavones (such as tangeretin and nobiletin) [7]. Previous studies have demonstrated that citrus flavonoids or citrus extracts have various beneficial biological functions in ruminants, such as reducing rumen inflammation [8], decreasing ruminal methane production [9], and improving milk′s oxidative stability [10]. Our latest study also showed that supplementing citrus peel extract (CPE) rich in flavonoids to the diet of cows decreased milk somatic cell count (SCC) and improved milk yield and milk lactose percentage [11]. However, the health-promoting effects associated with altered mammary microbiota and metabolites remain unknown.

Early studies compared the milk microbiome composition of dairy cows in health and mastitis, leading to an emerging view that mastitis occurs when the mammary microbiota is imbalanced rather than the result of a single pathogen′s action [12,13,14]. Wang et al. [4] reported that dietary supplementation with inulin altered the milk microbiota community in dairy cows. One study also found that adding bamboo leaf extract rich in flavonoids to the diet of cows decreased the relative abundances of *Corynebacterium_1*, *Aerococcus*, and *Staphylococcus* [5]. The milk constituents of dairy cows are influenced by many factors, including the lactation stage and cow health status [15]. Metabolomics-based approaches provide a rational way to display the overall metabolic response and dynamic changes in the mammary gland of dairy cows [16]. Sundekilde et al. [17] reported that milk concentrations of lactate, butyrate, isoleucine, acetate, and β-hydroxybutyrate were highly associated with the mammary health status in dairy cows.

Collectively, the bacterial composition and metabolites in the milk can not only reflect mammary health but also be associated with milk quality. Based on these studies, we hypothesized that supplementation of CPE could modulate milk microbiome and metabolites profile to improve mammary metabolic health. Therefore, the current study aimed to determine the effects of CPE supplementation on milk biochemical parameters, milk bacterial composition, and milk metabolites of lactating dairy cows. The 16S rRNA sequencing and liquid chromatography-tandem mass spectrometry (LC-MS)-based untargeted metabolomics was used to analyze the profiles of milk bacterial community and metabolites.

## 2. Materials and Methods

All procedures involving animals used in this study were approved by the Animal Care Committee of Beijing University of Agriculture (protocol no. BUA2021146; Beijing, China).

### 2.1. Citrus Peel Extract Preparation

The product CPE was provided by Shaanxi Xiazhou Biotechnology Co., Ltd. (Xi’an, China). According to the manufacturer, citrus flavonoids were extracted and enriched from the peel powder of *Citrus reticulata Blanco*, and the preparation was performed as previously reported [11]. The total flavonoid content of CPE was determined using commercially available kits (Beijing Boxbio Science & Technology Co., Ltd., Beijing, China), and absorbance 510 nm was recorded with rutin equivalents. A high-performance liquid chromatography system (1290 Infinity; Agilent Technologies, Inc., Santa Clara, CA, USA) was used to analyze the concentrations of major flavonoids in CPE, including naringin, hesperidin, neohesperidin, nobiletin, and tangeretin according to the method of Jiang et al. [18]. The chemical composition of CPE is presented in Table S1.

### 2.2. Animals, Experimental Design, and Treatments

Eight multiparous Chinese Holstein cows (662 ± 57.1 kg of body weight, 160 ± 22.4 days in milk, 36.1 ± 3.79 kg/d of milk production) were randomly allocated to four dietary treatments in a replicated 4 × 4 Latin square design balanced for residual effects. The four treatments included the basal diet (CON) and the basal diet supplemented with CPE at 50 g/d (CPE50), 100 g/d (CPE100), and 150 g/d (CPE150). There were four 21-d periods after 14 d of adaptation to diets, and the last 7 d were used for data collection. The CPE dosage for dairy cows used in the present study was determined according to previous animal studies done by our research group with plant extracts [19,20]. Animals were housed in a tie-stall barn equipped with a feed intake monitoring system. All cow diets were prepared in the morning and fed once daily for ad libitum intake as a total mixed ration (TMR; target of 5% refusal). Feed ingredients and nutritional levels are shown in Table S2. Cows received the TMR at 800 h and had continuous access to fresh drinking water. Cows were milked three times daily at approximately 600, 1400, and 2200 h, with yields recorded at each milking using an integrated milk meter (AfiMilk: SAE Afikim, Afikim, Israel). The CPE was top-dressed daily by mixing with approximately 500 g of TMR. We monitored the animals after feeding to ensure that the extracts were completely consumed.

### 2.3. Milk Sample Collection and Biochemical Parameters

Four 50-mL aliquot milk samples were collected from each cow at each milking on the last day of each period. The milk samples of each cow were composited in a 4:3:3 ratio for 3 milking times per day [21]. One composited milk sample was placed in a vial containing preservative (2-bromo-2-nitropropane-1,3-diol) and stored at 4 °C until being shipped to the Beijing Dairy Cows Center (Beijing, China) for milk component analyses. The data on milk yield and milk composition (fat, protein, lactose, SCC, and urea) was previously published [11]. Another composited sample was kept at −20 °C until the determination of immune-related indexes. The other two were placed in sterile tubes and stored at −80 °C to analyze the milk microbiome and metabolome.

For biochemical parameters analysis, 10 mL milk sample was centrifuged at 2500× *g* for 15 min at 4 °C to obtain skimmed milk. After the centrifugation at 12,000× *g* for 30 min at 4 °C, the supernatant was collected. Then, 100 μL of supernatant was taken and analyzed for the concentration of IgA, IgG, tumor necrosis factor-α (TNF-α), interleukin (IL)-1β, IL-6, IL-8, and interferon (IFN)-γ with commercially ELISA kits (Beijing Solarbio Science & Technology Co., Ltd., Beijing, China) according to the manufacturer’s instructions. Absorbance was recorded using a microplate reader (Multiskan FC; Thermo Fisher, New York, NY, USA). The 1,1-diphenyl-2-picrylhydrazyl radical (DPPH) scavenging activity, catalase (CAT) activity, malondialdehyde (MDA) content, and lysozyme (LZM) activity in milk supernatant (500 μL) were determined with the commercial kits (Nanjing Jiancheng Bioengineering Institute, Nanjing, China) following the manufacturer′s instructions. Absorbance was recorded by a spectrophotometer (752N, Shanghai Jingke Scientific Instrument Co., Ltd., Shanghai, China).

### 2.4. Milk 16S rRNA Gene Sequencing and Bioinformatic Analysis

Samples of CON and CPE150 were used for milk 16S rRNA gene sequencing. Frozen milk samples were thawed at 4 °C. One milliliter of milk was centrifuged for 10 min at room temperature at 16,100× *g* in an Eppendorf 5415R centrifuge. The supernatant was discarded, and the remaining pellet was resuspended in 400 μL of nuclease-free water. Microbial genomic DNA of each milk sample was extracted using the standard protocol for the E.Z.N.A.^®^ Soil DNA Kit (Omega Bio-tek, Norcross, GA, USA), except that 400 mg of lysozyme was added to the bacterial suspension and incubated for 12 h at 56 °C to maximize bacterial DNA extraction. The final DNA concentration and purity were evaluated with a NanoDrop 2000 spectrophotometer (Thermo Fisher Scientific, Waltham, MA, USA) and 1% agarose gel electrophoresis. The V3 to V4 region of the bacteria 16S rRNA gene was amplified by PCR (30 cycles of 95 °C for 30 s, 55 °C for 30 s, and 72 °C for 1 min; and final elongation at 72 °C for 5 min) using primers 338F (5′-barcode-ACTCCTRCGGGAGGCAGCAG-3′) and 806R (5′-GGACTACCVGGGTATCTAAT-3′), with the barcodes representing eight-base sequences unique to each sample. All PCR reactions were performed in triplicate in a total reaction volume of 20 μL, containing 2 μL of 2.5 mM dNTPs, 0.8 μL of each primer (5 mM), 0.4 μL of TransStart FastPfu DNA polymerase, 10 ng of DNA template, and distilled deionized water. Then PCR products were purified with an AxyPrep DNA gel extraction kit (Axygen, Biosciences, Union City, CA, USA). The sequencing libraries were pooled in equimolar concentrations. Amplicon sequencing (2 × 300 bp) was conducted at an Illumina MiSeq sequencing system (Illumina, San Diego, CA, USA). The sequencing data for our samples have been deposited into the Sequence Read Archive of NCBI under accession number PRJNA884519.

The paired-end reads were merged using FLASH (version 1.2.11; https://ccb.jhu.edu/software/FLASH/index.shtml; accessed on 10 September 2022). High-quality sequences were clustered into operational taxonomic units (OTU) at 97% sequence similarity using UPARSE (version 11). Representative OTU sequences were aligned and taxonomically classified using the RDP classifier (version 2.13; https://www.arb-silva.de/; accessed on 10 September 2022) against the SILVA database (version 138; https://www.arb-silva.de/; accessed on 10 September 2022). Alpha diversity indices were calculated using Mothur (version 1.30.2; https://www.mothur.org/wiki/Download_mothur; accessed on 12 September 2022) to evaluate the richness and diversity of the milk bacterial community between the two treatments. Beta diversity was analyzed with principal coordinate analysis (PCoA) based on Bray–Curtis and Abund–Jaccard distances using QIIME (version 1.9.1; http://qiime.org/install/index.html; accessed on 12 September 2022). The functional features of each sample were predicted using the Phylogenetic Investigation of Communities by Reconstruction of Unobserved States 2 (version 2.3.0-b; https://github.com/picrust/picrust2/; accessed on 15 September 2022). The difference in relative abundances of bacterial taxa and KEGG pathways at level 3 was evaluated by STAMP software (version 2.1.3) with Welch’s two-sided *t*-test. The obtained *p*-values were corrected for multiple comparisons using the Benjamini–Hochberg method.

### 2.5. Metabolomics Analysis

The milk samples used for metabolomics were the same as those used for microbiome sequencing. The milk sample preparation was conducted according to the protocol of Wang et al. [4]. The metabolites in the milk extracts were analyzed using a 1290 ultra-high performance liquid chromatograph system (Waters Corp., Milford, CT, USA) coupled to a Sciex 5600 TripleTOF^®^ (quadrupole-time-of-flight) MS system (AB SICEX, Framingham, MA, USA). The LC separation was performed with a UPLC BEH Amide column (100 mm × 2.1 mm × 1.7 μm) with mobile phase A (0.1% formic acid in water) and mobile phase B (0.1% formic acid in acetonitrile). The elution gradient was 5% B for 0–2 min, 5–95% B for 2–12 min, 95 % B for 12–15 min, and 95–5% B for 15–17 min. The flow rate was 0.40 mL/min. The column oven was set at 40 °C. The injection volume was 2 μL. The optimal conditions were set as follows: source temperature, 500 °C; curtain gas, 30 psi; both ion source GS1 and GS2, 50 psi; ion-spray voltage floating, −4000 V and 5000 V in negative and positive mode, respectively; declustering potential, 80 V; collision energy, 20 to 60 V rolling for MS/MS.

Raw LC-MS/MS data were imported into the Progenesis QI software (version 2.4; Nonlinear Dynamics Ltd., Newcastle, UK). The metabolites in these harvested data were annotated according to the accurate molecular weight (MW) by searching the exact MW against the Kyoto Encyclopedia of Genes and Genomes (KEGG) and Human Metabolome Database (HMDB). Principal component analysis (PCA) and supervised orthogonal partial least squares-discriminate analysis (OPLS-DA) were conducted in the SIMCA-P software (version 11.0; Umetrics AB, Umeå, Sweden). All OPLS models were tested for overfitting with a 200-time permutation test. The R^2^X and R^2^Y represent the cumulative modeled variation in variables X and Y. The Q^2^ is an estimate of the model’s predictive power, calculated by a cross-validation procedure. The differentially expressed metabolites were evaluated by combining the variable importance in the projection (VIP > 1.5) and the corrected *p*-values < 0.05 via the Student’s *t*-test. Differentially expressed metabolites were entered into the KEGG pathway analysis in MetaboAnalyst 5.0 (https://www.metaboanalyst.ca/; accessed on 18 September 2022) to determine possible involved pathways.

### 2.6. Statistical Analyses

Data on milk biochemical indicators were analyzed using the MIXED procedure of SAS (version 9.4; SAS Institute Inc., Cary, NC, USA) with the model as previously described [11]. The linear and quadratic effects of dietary CPE addition were assessed by orthogonal polynomial contrasts. All data presented in Table 1 are expressed as least squares means ± standard error of the means (SEM). Significance was declared when *p* ≤ 0.05, and a tendency was considered when 0.05 < *p* < 0.10. Spearman’s rank correlation analysis among significantly abundant milk bacteria, altered milk metabolites, and milk phenotype parameters was conducted using R (version 4.2.1) software. Only statistically significant (*p* < 0.05) and |r| > 0.5 can be considered a strong correlation.

## 3. Results

### 3.1. Milk Biochemical Parameters

As shown in Table 1, dietary supplementation with CPE did not affect (*p* > 0.05) milk IgA, IgG, TNF-α, IL-8, IFN-γ, or CAT. Milk IL-6 (*p* = 0.006) and MDA (*p* = 0.003) were linearly decreased with increasing CPE levels in the diet. CPE had a quadratic effect on milk IL-1β (*p* = 0.011), with the minimum value in CPE100 cows. Milk DPPH scavenging activity (*p* = 0.006) and LZM (*p* = 0.017) were linearly increased with increasing CPE amounts. Milk IL-8 concentration tended to reduce in a linear manner (*p* = 0.088) with the addition of CPE.

### 3.2. Milk Bacterial Community

**Figure 1:**
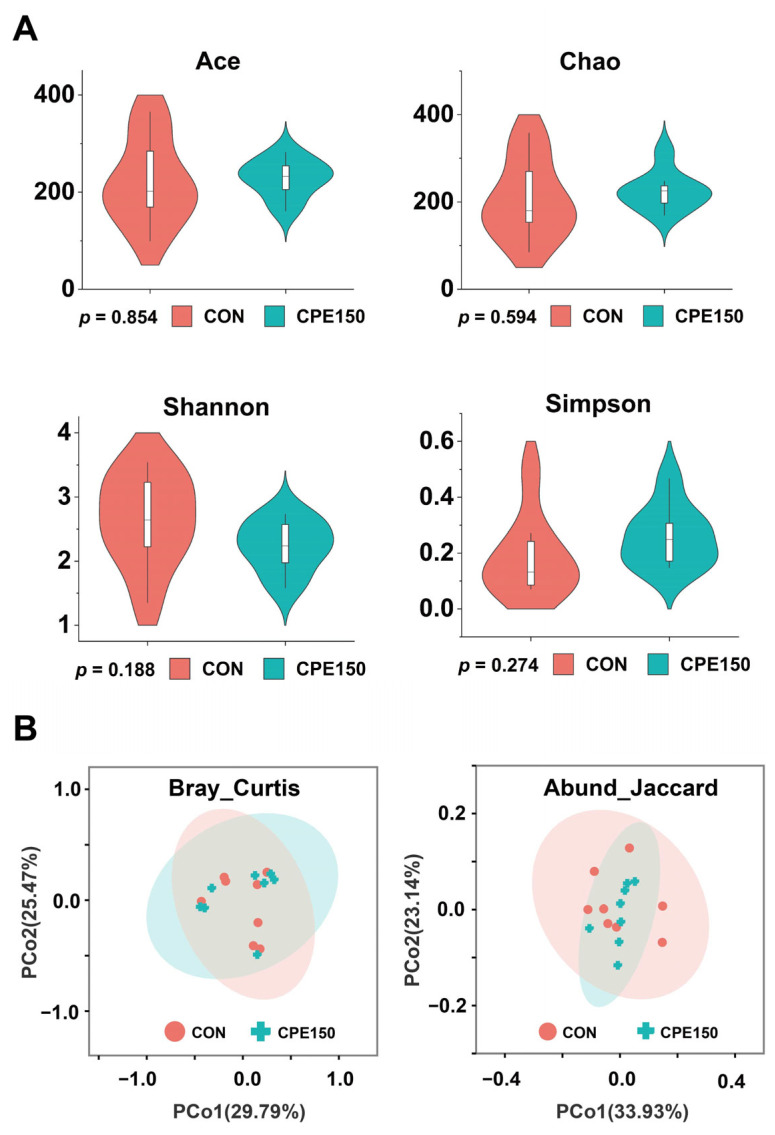
Comparison of the bacterial diversity between the milk of cows fed the control diet (CON), and supplementary citrus peel extracts at 150 g/d (CPE150). (**A**) Alpha diversity indices, including Ace, Chao, Shannon, and Simpson; (**B**) PCoA plots based on the Bray–Curtis and Abund–Jaccard metrics.

The amplicon sequencing of 16 milk samples generated a total of 67,199 high-quality reads, with an average of 42,450 sequences per sample. The average sequencing read length was 425 bp. Based on 97% sequence similarity, sequences were clustered into 467 OTU. A total of 331 OTU were shared between the CON and CPE150 cows, indicating the presence of an extensive common microbiome (Figure S1A). The rarefaction curves displaying the sequencing depth approached the saturation plateau in each sample, which meant the sequencing depth was reasonable (Figure S1B). The alpha diversity indices (Chao1, Ace, Shannon, and Simpson) did not significantly differ between CON and CPE150 (Figure 1A). The PCoA plot based on Bray–Curtis or Abund–Jaccard distance metrics showed no clear separation between CON and CPE150 (Figure 1B).

**Figure 2:**
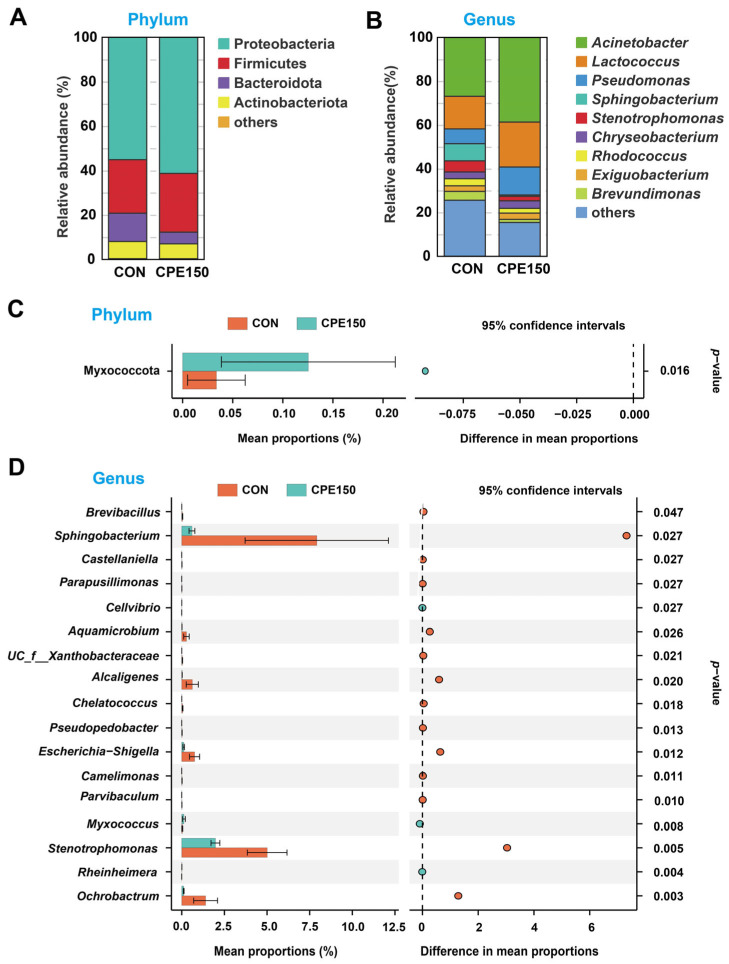
Comparison of the bacterial composition between the milk of cows fed the control diet (CON), and supplementary citrus peel extracts at 150 g/d (CPE150). Composition of milk bacteria at the phylum level (**A**) and genus level (**B**); Differential bacterial taxa at the phylum level (**C**) and genus level (**D**). A Wilcoxon rank-sum test was used to test differences in taxa abundance between two treatments (*n* = 8 per treatment).

The bacterial community composition at the phylum and genus level was evaluated (Figure 2A,B). At the phylum level, the milk microbiota was dominated by Proteobacteria, Firmicutes, Bacteroidota, and Actinobacteriota. At the genus level, the predominant taxa were *Acinetobacter*, *Lactococcus*, *Pseudomonas*, *Sphingobacterium*, *Stenotrophomonas*, and *Chryseobacterium*. Compared with CON, CPE150 cows had a greater abundance of phylum Myxococcota in the milk (Figure 2C). The relative abundances of 17 genera displayed significant differences (*p* < 0.05) between CPE150 vs. CON (Figure 2D). The abundances of 14 genera, including *Brevibacillus*, *Sphingobacterium*, *Castellaniella*, *Parapusillimonas*, *Aquamicrobium*, *unclassified_f__Xanthobacteraceae*, *Alcaligenes*, *Chelatococcus*, *Pseudopedobacter*, *Escherichia-Shigella*, *Camelimonas*, *Parvibaculum*, *Stenotrophomonas*, and *Ochrobactrum* were decreased in CPE150 cows in comparison with CON. The abundance of *Cellvibrio*, *Myxococcus,* and *Rheinheimera* increased significantly in the CPE150 cows compared with CON.

**Figure 3:**
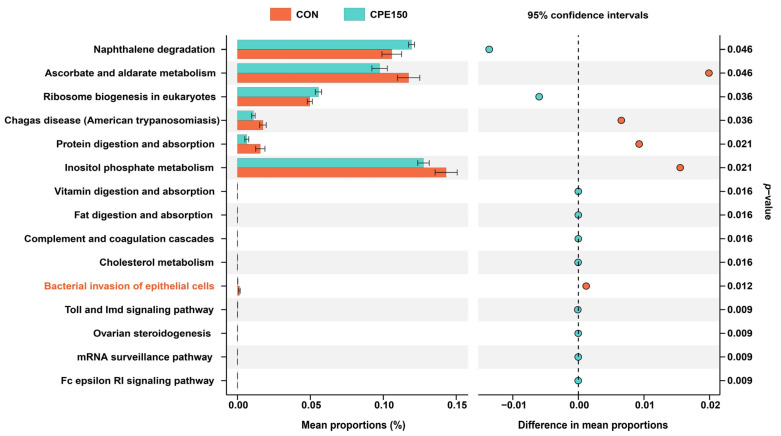
Differential predicted KEGG pathways at level 3 based on the milk bacterial sequences between dairy cows fed the control diet (CON) and supplementary citrus peel extracts at 150 g/d (CPE150). A Wilcoxon rank-sum test was used to test differences in taxa abundance between two treatments (*n* = 8 per treatment).

The significantly differential microbial functions at KEGG level 3 are shown in Figure 3. Compared with the CON, the relative abundances of ‘naphthalene degradation’, ‘ribosome biogenesis in eukaryotes’, ‘vitamin digestion and absorption’, ‘fat digestion and absorption’, ‘complement and coagulation cascades’, ‘cholesterol metabolism’, ‘toll and Imd signaling pathway’, ‘ovarian steroidogenesis’, ‘mRNA surveillance pathway’, and ‘Fc epsilon RI signaling pathway’ were greater in the CPE150 cows. However, the relative abundances of ‘ascorbate and aldarate metabolism’, ‘Chagas disease (American trypanosomiasis)’, ‘protein digestion and absorption’, ‘inositol phosphate metabolism’, and ‘bacterial invasion of epithelial cells’ were less in the CPE150 cows compared with the CON.

### 3.3. Milk Metabolomics Profiling

**Figure 4:**
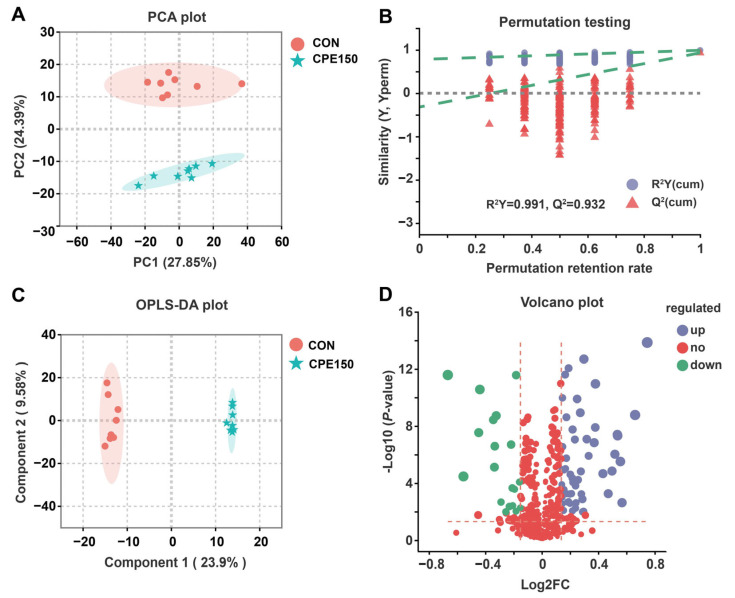
Milk metabolome analysis of dairy cows fed the control diet, and supplementary citrus peel extracts at 150 g/d (CPE150). (**A**) Unsupervised PCA of milk metabolites. (**B**) Permutation testing of the supervised orthogonal partial least squares-discriminate analysis (OPLS-DA) model; Q^2^, predictive ability parameter; R^2^Y, goodness-of-fit parameter. (**C**) OPLS-DA scatter plot. (**D**) Volcano plot of differential metabolites; Blue dots (‘up’) and green dots (‘down’) indicate up-regulated and down-regulated metabolites (CPE150 vs. CON), respectively; red dots (‘no’) indicate metabolites for which there was no significant difference (CPE150 vs. CON); FC, fold change.

After quality control, a total of 833 metabolites with known identities were obtained against KEGG, HMDB, and the self-built database of Majorbio Company (Shanghai, China). The scores plot of the PCA, an unsupervised multivariate data analysis method, presented clear discrimination between the milk metabolome of the CON and CPE150 (Figure 4A). The validity of the OPLS-DA model was verified through a permutation test (200 permutations). The OPLS-DA model parameters R^2^Y and Q^2^ were 0.977 and 0.932, indicating that the OPLS-DA model had good fitness and predictive ability (Figure 4B). The intercept value of Q^2^ was −0.3320 at a threshold <0, suggesting that the OPLS-DA model was not overfitted. The OPLS-DA plot also showed distinct separations for milk metabolites between the CON and CPE150 (Figure 4C).

**Figure 5:**
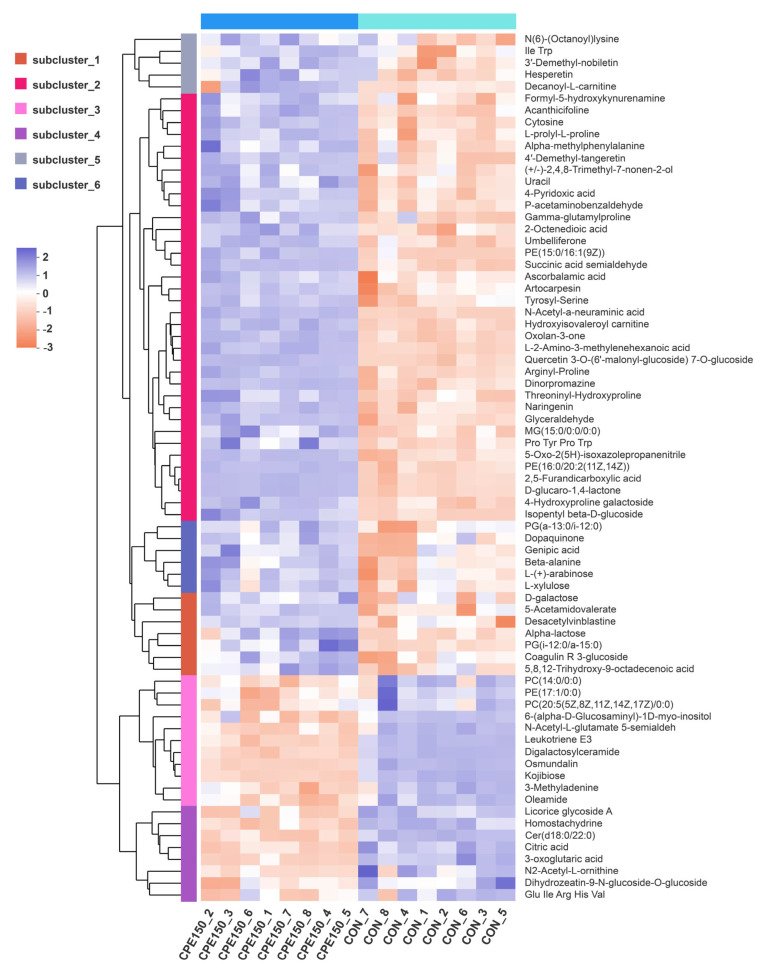
Clustering heatmap of differentially expressed metabolites between the milk of dairy cows fed the control diet (CON) and supplementary citrus peel extracts at 150 g/d (CPE150).

**Figure 6:**
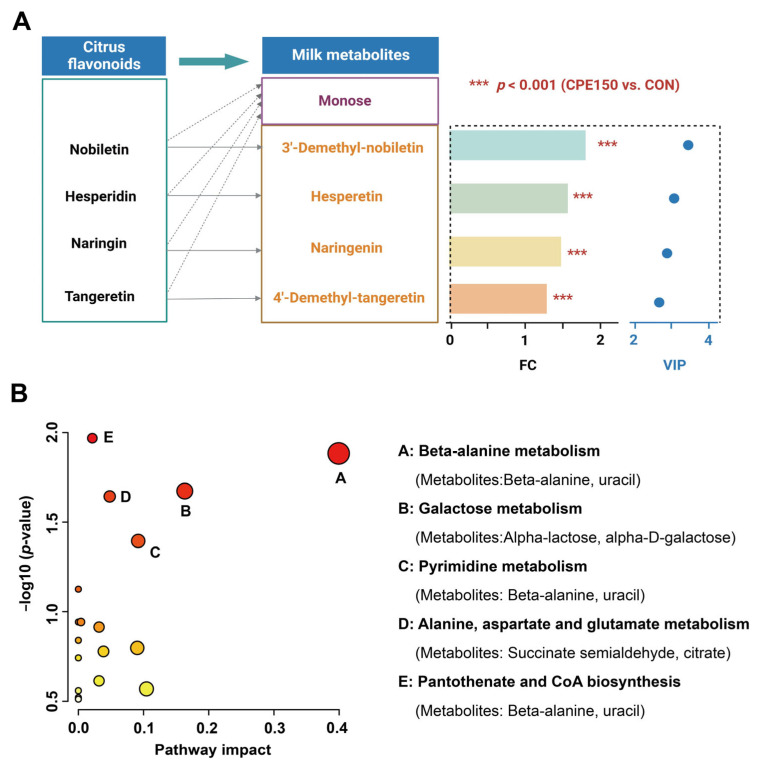
Key differentially expressed metabolites analysis. (**A**) The aglycones of flavonoids were enriched in the milk of dairy cows fed the supplementary citrus peel extracts at 150 g/d (CPE150) in comparison with the control diet (CON); Blue points indicate VIP values of metabolites (**B**) Overview of pathway analysis based on the differentially expressed metabolites between the milk of dairy cows fed the CON vs. CPE150. FC, fold change; VIP, variable importance in the projection.

Differential milk metabolites were screened by VIP > 1.5, fold change (FC) > 1.1 or FC < 0.9, and corrected *p*-value < 0.05. A total of 73 differentially expressed metabolites were obtained (Figure 4D). Among them, 54 metabolites, such as N-Acetyl-a-neuraminic acid, succinic acid semialdehyde, desacetylvinblastine, and threoninyl-hydroxyproline, were enriched in the milk of the CPE150 cows (Figure 5 and Table S3). However, the CON cows had greater concentrations of 19 metabolites in milk, such as osmundalin, licorice glycoside A, digalactosylceramide, leukotriene E3, 3-Oxoglutaric acid, and Cer(d18:0/22:0) in the milk. The milk metabolomic data showed that the metabolite of main dietary citrus flavonoids, including 3′-demethyl-nobiletin, hesperetin, naringenin, and 4′-demethyl-tangeretin, were enriched in the milk of the CPE150 cows (Figure 6A). Metabolic pathway analysis based on 73 differentially expressed metabolites displayed the significant enrichment of five pathways (*p* < 0.05), including ‘beta-alanine metabolism’ (impact = 0.40), ‘galactose metabolism’ (impact = 0.16), ‘pyrimidine metabolism’ (impact = 0.09), ‘alanine, aspartate and glutamate metabolism’ (impact = 0.05), and ‘pantothenate and CoA biosynthesis’ (impact = 0.02). Other metabolic pathways (*p* > 0.05) with low impacts could be neglected.

### 3.4. Correlation Analysis

**Figure 7:**
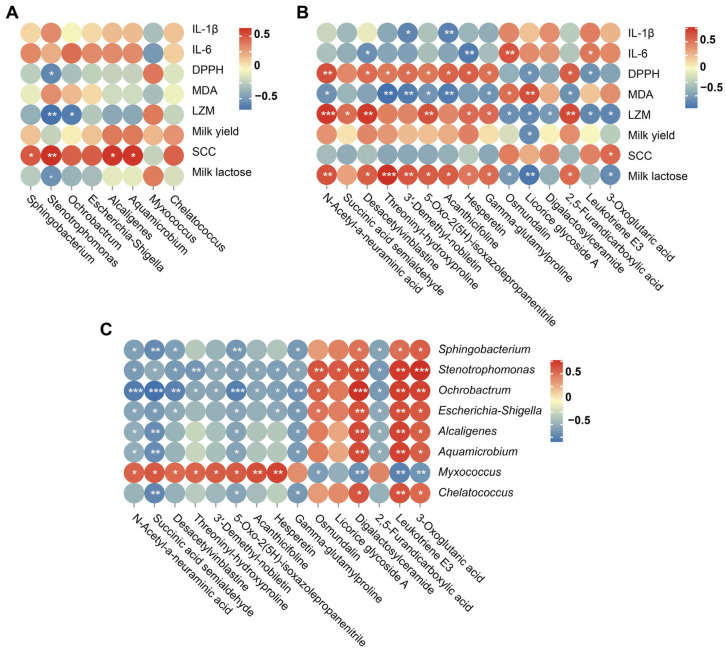
Spearman’s correlation heatmap. (**A**) Correlation analysis between significantly differential milk phenotype parameters and differentially abundant milk bacterial taxa at the genus level; (**B**) Correlation analysis between significantly differential milk phenotype parameters and differentially expressed metabolites; (**C**) Correlation analysis between differentially abundant milk bacterial taxa at the genus level and differentially expressed metabolites. IL-1β, interleukin-1β, IL-6, interleukin-6; DPPH, 1,1-Diphenyl-2-picrylhydrazyl radical; MDA, malondialdehyde; LZM, lysozyme; SCC, somatic cell count. * *p* < 0.05, ** *p* < 0.01, *** *p* < 0.001.

Spearman’s correlation analysis among significantly differential milk phenotypes, milk bacterial genus (top 8), and milk metabolites (top 15) are shown in Figure 7A–C. Milk DPPH scavenging activity was negatively correlated with *Stenotrophomonas*. Milk LZM was negatively correlated with *Ochrobactrum* and *Stenotrophomonas*. Milk SCC was positively correlated with *Stenotrophomonas*, *Sphingobacterium*, *Alcaligenes*, and *Aquamicrobium*. Milk lactose percentage was negatively correlated with *Stenotrophomonas*. Milk DPPH scavenging activity, LZM, and lactose were positively correlated with some up-regulated metabolites while negatively correlated with down-regulated metabolites. In addition, genera *Stenotrophomonas*, *Ochrobactrum,* and *Myxococcus* presented strong correlations with differentially expressed milk metabolites.

## 4. Discussion

In dairy ruminants, the health of the mammary gland is crucial to produce high-quality milk [22]. It has been demonstrated that plant extracts or phytochemicals exert their preventative and protective effects on the bovine mammary gland against infection [4,5,19,23]. Our previous study showed that dietary CPE addition reduced milk SCC and increased milk yield [11], indicating that citrus flavonoids could improve the immunometabolism status in the bovine mammary. Microbiota and the immune system are closely linked to the development and maintenance of host health, as recently demonstrated [24]. Therefore, we analyzed the immune and antioxidant indices, microbiome, and metabolomics to understand the mechanisms responsible for the effects of CPE on mammary health.

As immunoregulatory components of milk, immunoglobulins and LZM work individually, additively, and synergistically to target and deactivate pathogens [25,26]. In addition to acting as immune regulators, these compounds protect udders against pathogenic and opportunistic microorganisms that cause intramammary infections [27]. In the present study, supplementing CPE increased milk LZM content, suggesting that the CPE contributes indirectly to the mammary defense system against infection. In addition, we detected lower levels of proinflammatory factors IL-1β and IL-6 when cows received CPE. The results suggested that CPE could exert its anti-inflammation effect on the mammary gland of dairy cows. The effect was in line with the observations of Burmanczuk et al. [28], who found that intramammary administration of hesperidin and naringenin decreased milk SCC of dairy cows with mastitis.

Flavonoids have powerful antioxidant properties in vivo and can scavenge a wide variety of reactive oxygen, nitrogen, and chlorine species [29]. Previous studies reported that antioxidant activity in milk was improved significantly in cows fed diets supplemented with flavonoids [30,31]. As a marker of oxidative stress, MDA can indicate a counterbalance to high levels of oxidative phosphorylation [32]. The DPPH is a relatively stable organic radical widely used to test samples for its ability to scavenge free radicals [33]. The improved DPPH scavenging activity and decreased MDA content were observed in the milk of cows who received supplementary CPE, suggesting that CPE has the potential to enhance the milk antioxidants. Furthermore, strong correlations were observed between flavonoid metabolites (3′-demethyl-nobiletin and hesperetin) and DPPH scavenging activity. These findings indicated that flavonoid metabolites derived from CPE could be transferred and enriched in the mammary epithelial cells and milk to exert their biological activity.

The milk microbiota composition has been shown to have important implications for lactating dairy cows’ mammary health [12,34]. In line with previous studies [5,24], the taxonomic profiles of milk were dominated by Proteobacteria, Firmicutes, Bacteroidota, and Actinobacteria. Although these dominant taxa in the phylum level were not different between the two groups, the relative abundance of Myxococcota was increased in the CPE150 cows. To the best of our knowledge, no information regarding the ecological niches of Myxococcta in bovine milk is available. Bacteria of Myxococcota utilize large quantities of viable microbes and biological macromolecules by producing secondary metabolites and extracellular enzymes, and the predator’s pattern was considered the main way to obtain energy and nutrients [35]. It was reported that the bacteria are typically present in small amounts in the human gut [36]. However, the reason for the increased Myxococcota response to CPE was not entirely clear, and further research is required.

At the genus level, species belonging to *Stenotrophomonas*, *Streptococcus*, *Staphylococcus Pseudomonas*, and *Alcaligenes* were considered as main pathogenic and opportunistic bacteria in the mammary [34]. Among them, intramammary infections with *Streptococcus agalactiae* and *Staphylococcus aureus* are major causes of bovine mastitis [37]. However, no difference in the relative abundance of *Streptococcus* or *Staphylococcus* was found between the CON and CPE150. The relative abundances of *Stenotrophomonas* and *Alcaligenes* were less in the CPE150 compared with the CON. It was reported that *Stenotrophomonas* was more predominant in the milk of cows with clinical mastitis [38]. In addition, a *Stenotrophomonas* isolate has been demonstrated to be involved in keratin degradation [39]. Considering that one of the major innate immune mechanisms of the bovine mammary gland is the production of keratin plugs over the teat canal, the ability to degrade keratin would likely enhance the ability of *Stenotrophomonas* to colonize mammary [40].

The bacteria within *Escherichia-Shigella* have been reported to exist in human or animal gut, where their enrichment contributes to the development of intestinal disease and inflammation [41]. Similarly, Wang et al. [4] reported that dietary supplementation with inulin inhibited *Escherichia-Shigella* in the milk of dairy cows with subclinical mastitis. Bacteria within *Sphingobacterium* contain large amounts of sphingophospholipid compounds in their cell membranes [5]. It was reported that *Sphingobacterium* species are intrinsically resistant to many commonly employed antibiotics [42]. Oikonomou et al. [13] reported that the *Sphingobacterium* prevalence was positively correlated with SCC, and species of *Sphingobacterium* may also be opportunistic pathogens. Additionally, in the current experiment, the genera *Ochrobactrum* was decreased due to CPE supplementation. *Ochrobactrum anthropi* was recognized as an opportunistic pathogen in human milk [43]. However, the relationship between the abundance of *Ochrobactrum* species and bovine mastitis has not been reported previously, and it needs further investigation.

Positive correlations between milk SCC and the abundances of *Stenotrophomonas*, *Sphingobacterium*, or *Alcaligenes* may also suggest that CPE could decrease SCC via modulating the mammary microbial community. In addition, we also observed that the functional feature ‘bacterial invasion of epithelial cells’ was decreased by CPE. The functional differences between the two groups may provide clues to further evaluation of the health of the bovine mammary glands in response to CPE addition.

Cold storage after milk collection and during the transportation is vital for maintaining raw milk quality [34]. It is well known that psychrotrophic bacteria are spoilage-causing bacteria in raw milk, and they cause significant economic losses to the dairy industry [44]. Species of *Sphingobacterium* mainly exhibit lipolytic activity, although some strains exhibit proteolytic activity as well [45]. Furthermore, Gram-negative bacteria such as *Alcaligenes* spp. also cause the spoilage of dairy products [45]. Therefore, the decreased abundances of *Sphingobacterium* and *Alcaligenes* in the present study could contribute to the improvement in the organoleptic quality of raw milk and dairy products.

The metabolites present in milk can originate from several different sources, including being transported from the bloodstream, released from damaged somatic cells, or actively secreted from bacteria and mammary epithelial cells [17]. It has been documented that metabolite concentrations in the milk could be used as biomarkers to monitor the health of cows [16]. The somatic cells of milk contain different cell types, which may contribute to the different metabolite fingerprints of milk [17].

As typically observed in the large intestine of monogastric, rumen microorganisms can cleave the glycosidic components of polymeric flavonoids, thus contributing to the intestinal absorption of flavonoid compounds or their metabolites [46]. For instance, the active aglycone forms naringenin and hesperetin are required to exert various health-promoting activities in the mammary gland. As expected, the metabonomic analysis showed that some key metabolites of citrus flavonoids were enriched in the milk of dairy cows, indicating these metabolites played crucial roles in improving mammary health.

Notably, beta-alanine played a central role in several related metabolic pathways, indicating a key role in this compound. It is believed that most beta-alanine metabolism occurs in the brain and muscles, and the final product of normal metabolism is acetic acid [47]. Meanwhile, beta-alanine can maintain the antioxidant capacity of the body [48], and the increase in beta-alanine could be an indicator of improved milk quality. Similarly, Hou et al. [19] reported that the milk of cows that received the artemisinin had a greater concentration of beta-alanine. However, little information is available about the relationship between flavonoids and beta-alanine metabolism.

Leukotriene E3 is considered a proinflammation compound in various diseases [49]. In accordance with our results, Wang et al. [4] reported that supplementation with inulin reduced the concentration of milk leukotriene E3 in dairy cows with mastitis. In the present study, supplementary CPE caused a decrease in milk Cer(d18:0/22:0) and digalactosylceramide. As structural membrane constituents and essential eukaryotic signaling molecules, Sphingolipids play an important role in modulating immunity and inflammation status [50]. Ceramides, as mediators of oxidative stress and inflammation, are widely perceived to be the main biologically active sphingolipids [51]. Thus, it is tempting to speculate that the fewer concentrations of leukotriene E3, Cer(d18:0/22:0), and digalactosylceramide reflected lower inflammation levels in the mammary gland. We also found that the D-galactose and alpha-lactose were increased by CPE addition, and the metabolic pathway ‘galactose metabolism’ was enriched. The results might be associated with increased milk lactose synthesis, which was reported in our previous study [11].

## 5. Conclusions

We found that dietary CPE decreased milk proinflammation factor levels, increased milk antioxidant ability, and altered the profiling of milk bacteria and metabolites, which mediated the health-promoting effects of CPE on the mammary gland. Dietary CPE with abundant flavonoids can transfer flavonoid metabolites, such as 3′-demethyl-nobiletin, hesperetin, and naringenin, to the mammary and milk. These compounds exert antioxidant and anti-inflammatory activities in the mammary. The consumption of CPE inhibited the proliferation of opportunistic pathogens, which could decrease the risk of mastitis by maintaining the ecological balance of the mammary microbiota. In short, dietary supplementation with CPE could be a promising strategy for improving mammary health and enhancing product quality. Future studies should aim to investigate the dynamic changes in milk microbiome and metabolites profiling during the adaptation to CPE metabolism to identify the biomarkers of mammary health in dairy cows.

## Figures and Tables

**Table 1:** Effects of feeding citrus peel extract (CPE) on biochemical parameters of milk from dairy cows.

Item ^1^	CPE Supplementation, g/d				SEM	*p*-Value ^2^		
	0	50	100	150		T	L	Q
IgA, mg/mL	0.96	1.04	1.03	1.03	0.037	0.277	0.168	0.241
IgG, mg/mL	4.38	4.39	4.64	4.59	0.161	0.476	0.185	0.844
TNF-α, pg/mL	79.25	84.61	83.17	84.61	3.780	0.693	0.385	0.601
IL-1β, pg/mL	71.10 ^a^	65.87 ^b^	65.28 ^b^	67.20 ^b^	1.240	0.010	0.045	0.011
IL-6, pg/mL	165.32 ^a^	166.66 ^a^	153.22 ^b^	153.88 ^b^	3.241	0.008	0.006	0.925
IL-8, pg/mL	67.65	65.20	63.37	64.99	1.262	0.117	0.088	0.112
IFN-γ, pg/mL	47.33	44.31	46.22	45.00	1.050	0.178	0.314	0.422
DPPH scavenging activity, %	19.86 ^b^	20.27 ^b^	22.59 ^ab^	23.39 ^a^	0.954	0.030	0.006	0.841
CAT, U/mL	215.69	216.27	216.03	214.19	1.970	0.867	0.599	0.550
MDA, ng/mL	8.56 ^a^	8.23 ^ab^	8.02 ^b^	7.84 ^b^	0.164	0.031	0.017	0.714
LZM, μg/mL	10.87 ^b^	11.53 ^ab^	13.43 ^a^	13.54 ^a^	0.756	0.020	0.003	0.697

^1^ IgA, immunoglobulin A; IgG, immunoglobulin G; TNF-α, tumor necrosis factor-α; IL-1β, interleukin-1-1β, IL-6, interleukin-6; IL-8, interleukin-8; IFN-γ, interferon-γ; DPPH, 1,1-Diphenyl-2-picrylhydrazyl radical; CAT, catalase; MDA, malondialdehyde; LZM, lysozyme. ^2^ T, treatment; L, linear; Q, quadratic. ^a,b^ Means within a row with different superscripts differ (*p* < 0.05).

## Data Availability

The sequencing data for milk bacteria have been deposited into the Sequence Read Archive of NCBI under accession number PRJNA884519.

## Supplementary Materials

The following supporting information can be downloaded at: https://www.mdpi.com/article/10.3390/foods11244119/s1. Table S1: Chemical composition of the citrus peel extracts; Table S2: Ingredients and chemical composition of the basal ration; Table S3: Identification of significant differentially expressed metabolites in the milk of dairy cows by comparison of the CON and CPE150 with VIP > 1.5 and *p* < 0.05; Figure S1: Operation taxonomic unit (OTU) numbers of milk samples of dairy cows fed the CON and CPE150.
