# Supplementary material for: Microbiome-Metabolomics Insights into the Milk of Lactating Dairy Cows to Reveal the Health-Promoting Effects of Dietary Citrus Peel Extracts on the Mammary Metabolism
Source: Foods. 2022 Dec 19;11(24):4119. doi: 10.3390/foods11244119 (PMC9778193; doi:10.3390/foods11244119)
Supplement: Supplementary file 1 [file foods-11-04119-s001.zip › foods-2043952-supplementary.pdf]

Table S1. Chemical composition of the citrus peel extracts.

Item	Contents
Chemical composition, % of DM	
DM, % as fed	92.16
OM	85.37
Total flavonoids	38.38
Naringin	12.89
Hesperidin	19.52
Neohesperidin	1.22
Nobiletin	3.21
Tangeretin	2.07

DM = dry matter; OM = organic matter.

Table S2. Ingredients and chemical composition of the basal ration.

Item[1]	Contents
Ingredient, % of DM	
Corn silage	34.59
Alfalfa hay	11.85
Oat hay	4.33
Ground Corn grain	17.73
Flaked corn	3.98
Soybean meal	10.46
Canola meal	2.90
Sugar beet pulp	0.56
Whole cottonseed	7.12
Fat powder[2]	1.33
Mineral and vitamin mix[3]	3.87
Molasses	1.28
Chemical composition, % of DM[4]	
DM, % as fed	51.43
OM	91.12
CP	17.03
NDF	33.85
ADF	16.70
EE	4.96
Starch	25.69
NE_L (Mcal/kg of DM)[5]	1.66

[1]DM = dry matter; OM = organic matter; CP = crude protein; NDF = neutral detergent fiber; ADF = acid detergent fiber; EE = ether extract; NE_L = Net energy for lactation.

[2]Bergafat, a saturated free fatty acid supplement (Berg+Schmidt GmbH & Co. KG, Hamburg, Germany).

[3]Formulated to contain (as-is basis) trace mineral mix, 0.95%; dry corn distillers grains with solubles, 44.9%; MgO (56% Mg), 7.8%; NaCl, 6.6%; vitamin ADE premix, 0.48%; limestone, 39.2%; and selenium premix, 0.07%. Ca, 14.3%; P, 0.38%; Mg, 4.57%; K, 0.46%; S, 0.39%; Zn, 1,218 mg/kg; Fe, 186 mg/ kg, Se, 6.93 mg/kg; Cu, 370 mg/kg; vitamin A, 272,000 IU/kg; vitamin D, 75,000 IU/kg; and vitamin E, 2,080 IU/kg.

[4]Analyzed values.

[5]Calculated according to NRC (2001).

Table S3. Identification of significant differentially expressed metabolites in the milk of dairy cows by comparison of CON and CPE150 with VIP > 1.5 and *P* < 0.05.

Compounds	VIP[1]	FC[2]	Corrected *P*-value
**Up-regulated in the CPE150**			
N-Acetyl-a-neuraminic acid	4.18	1.68	< 0.05
Succinic acid semialdehyde	3.75	1.58	< 0.05
Desacetylvinblastine	3.32	1.47	< 0.05
Threoninyl-hydroxyproline	3.31	1.45	< 0.05
3′-Demethyl-nobiletin	3.30	1.58	< 0.05
5-Oxo-2(5H)-isoxazolepropanenitrile	3.25	1.30	< 0.05
Acanthicifoline	3.10	1.23	< 0.05
Hesperetin	3.02	1.53	< 0.05
Gamma-glutamylproline	3.00	1.41	< 0.05
2,5-Furandicarboxylic acid	2.98	1.35	< 0.05
Isopentyl beta-D-glucoside	2.93	1.29	< 0.05
Cytosine	2.92	1.39	< 0.05
Umbelliferone	2.80	1.21	< 0.05
Naringenin	2.87	1.47	< 0.05
4′-Demethyl-tangeretin	2.68	1.35	< 0.05
4-Hydroxyproline galactoside	2.67	1.30	< 0.05
D-glucaro-1,4-lactone	2.65	1.19	< 0.05
Ile Trp	2.64	1.25	< 0.05
Decanoyl-L-carnitine	2.41	1.16	< 0.05
5,8,12-Trihydroxy-9-octadecenoic acid	2.39	1.22	< 0.05
5-Acetamidovalerate	2.30	1.21	< 0.05
L-prolyl-L-proline	2.28	1.18	< 0.05
Tyrosyl-Serine	2.24	1.12	< 0.05
Hydroxyisovaleroyl carnitine	2.24	1.13	< 0.05
Dopaquinone	2.23	1.14	< 0.05
PG(a-13:0/i-12:0)	2.22	1.17	< 0.05
(+/-)-2,4,8-Trimethyl-7-nonen-2-ol	2.17	1.19	< 0.05
Glyceraldehyde	2.12	1.16	< 0.05
PG(i-12:0/a-15:0)	2.11	1.23	< 0.05
Dinorpromazine	2.10	1.21	< 0.05
PE(16:0/20:2(11Z,14Z))	2.09	1.15	< 0.05
Uracil	2.08	1.18	< 0.05
Ascorbalamic acid	2.03	1.13	< 0.05
Pro Tyr Pro Trp	2.02	1.11	< 0.05
Beta-alanine	1.94	1.11	< 0.05
L-(+)-arabinose	1.94	1.15	< 0.05
L-xylulose	1.91	1.18	< 0.05
L-2-amino-3-methylenehexanoic acid	1.89	1.16	< 0.05

Genipic acid	1.88	1.18	< 0.05
Formyl-5-hydroxykynurenamine	1.88	1.22	< 0.05
Arginyl-Proline	1.85	1.12	< 0.05
4-Pyridoxic acid	1.81	1.10	< 0.05
2-Octenedioic acid	1.81	1.11	< 0.05
Quercetin 3-O-(6'-malonyl-glucoside) 7-O-glucoside	1.81	1.11	< 0.05
Artocarpesin	1.79	1.10	< 0.05
P-Acetaminobenzaldehyde	1.77	1.16	< 0.05
N(6)-(Octanoyl)lysine	1.75	1.11	< 0.05
D-Galactose	1.73	1.13	< 0.05
Oxolan-3-one	1.73	1.15	< 0.05
Alpha-lactose	1.72	1.13	< 0.05
Coagulin R 3-glucoside	1.71	1.12	< 0.05
PE(15:0/16:1(9Z))	1.69	1.10	< 0.05
MG(15:0/0:0/0:0)	1.66	1.14	< 0.05
Alpha-methylphenylalanine	1.63	1.10	< 0.05
**Down-regulated in the CPE150**			
Osmundalin	3.82	0.63	< 0.05
Licorice glycoside A	3.60	0.68	< 0.05
Digalactosylceramide	3.33	0.74	< 0.05
Leukotriene E3	2.95	0.80	< 0.05
3-Oxoglutaric acid	2.94	0.73	< 0.05
Cer(d18:0/22:0)	2.83	0.79	< 0.05
N-Acetyl-L-glutamate 5-semialdeh	2.66	0.79	< 0.05
Homostachydrine	2.66	0.79	< 0.05
Kojibiose	2.55	0.88	< 0.05
Citric acid	2.55	0.86	< 0.05
6-(alpha-D-glucosaminyl)-1D-myo-inositol	2.16	0.86	< 0.05
PC(14:0/0:0)	2.04	0.85	< 0.05
N2-Acetyl-L-ornithine	1.97	0.82	< 0.05
Glu Ile Arg His Val	1.94	0.84	< 0.05
PC(20:5(5Z,8Z,11Z,14Z,17Z)/0:0)	1.82	0.87	< 0.05
3-Methyladenine	1.80	0.87	< 0.05
Oleamide	1.77	0.90	< 0.05
PE(17:1/0:0)	1.72	0.88	< 0.05
Dihydrozeatin-9-N-glucoside-O-glucoside	1.60	0.90	< 0.05

[1]VIP = variable importance in the projection.

[2]FC = fold change.

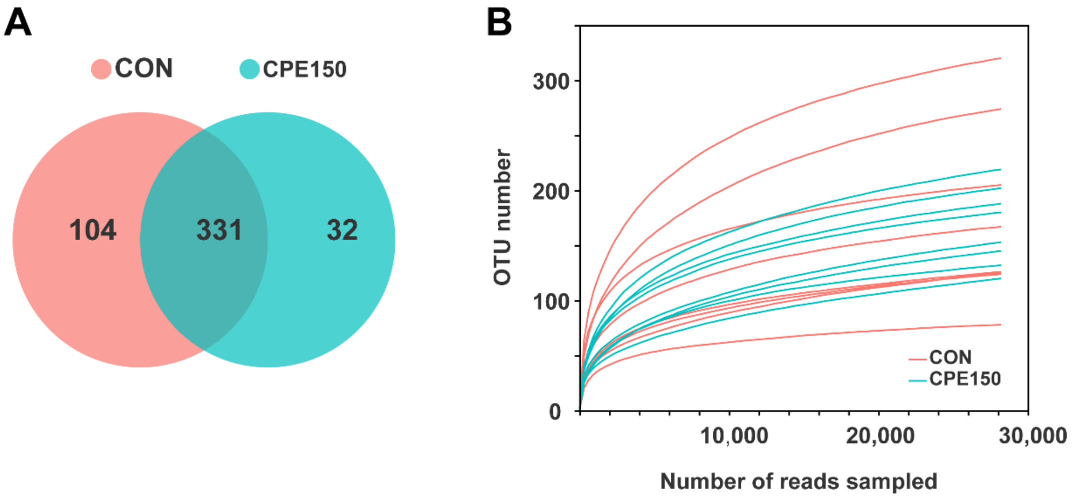

Figure S1. Operation taxonomic unit (OTU) numbers of milk samples of dairy cows fed the CON and CPE150. (A) Venn diagram; (B) Rarefaction curves.
